# *Staphylococcus aureus* Infections in New Zealand, 2000–2011

**DOI:** 10.3201/eid2007.131923

**Published:** 2014-07

**Authors:** Deborah A. Williamson, Jane Zhang, Stephen R. Ritchie, Sally A. Roberts, John D. Fraser, Michael G. Baker

**Affiliations:** University of Auckland, Auckland, New Zealand (D.A. Williamson, S.R. Ritchie, J.D. Fraser);; Institute of Environmental Science and Research, Wellington, New Zealand (D.A. Williamson);; University of Otago, Wellington (J. Zhang, M.G Baker);; Auckland District Health Board, Auckland (S.A. Roberts)

**Keywords:** Infectious disease epidemiology, skin infections, inequality, sepsis, New Zealand, Staphylococcus aureus, bacteria

## Abstract

The incidence rate for invasive and noninvasive *Staphylococcus aureus* infections in New Zealand is among the highest reported in the developed world. Using nationally collated hospital discharge data, we analyzed the epidemiology of serious *S. aureus* infections in New Zealand during 2000–2011. During this period, incidence of *S. aureus* skin and soft tissue infections increased significantly while incidence of staphylococcal sepsis and pneumonia remained stable. We observed marked ethnic and sociodemographic inequality across all *S. aureus* infections; incidence rates for all forms of *S. aureus* infections were highest among Māori and Pacific Peoples and among patients residing in areas of high socioeconomic deprivation. The increased incidence of *S. aureus* skin and soft tissue infections, coupled with the demographic disparities, is of considerable concern. Future work should aim to reduce this disturbing national trend.

## Abstract 2

Skin and soft tissue infections increased significantly; sociodemographic disparity was noted.

Despite advances in diagnostics and therapeutics, the clinical and economic burdens of *Staphylococcus aureus* infections remain a substantial public health problem (*1*). During the past decade in several parts of the world, most notably in North America, the epidemiology of *S. aureus* infections has changed dramatically, predominantly because of the epidemic spread of a strain of community-associated methicillin-resistant *S. aureus* (MRSA) (*2*,*3*). Infections caused by community-associated MRSA are most commonly skin and soft tissue infections (SSTIs) and typically occur in patients with no history of exposure to health care facilities (*1*). In addition, specific sociodemographic associations for community-associated MRSA infection have been described and include younger patient age, specific ethnic groups, and economic deprivation (*1*,*4*,*5*). Although the epidemiology of *S. aureus* infections has been well studied in North America, comparatively little is known about the trends and patient demographics for *S. aureus* infections in other geographic settings, particularly in the Southern Hemisphere. Knowledge of the overall prevalence and distribution of *S. aureus* infections, regardless of methicillin resistance, at a population level is crucial for informing prevention and control strategies.

The incidence of invasive and noninvasive *S. aureus* infections is reportedly higher In New Zealand than in other developed countries; rates are highest among Māori (indigenous New Zealanders) and Pacific Peoples (*6*–*9*). For example, in 1 study, *S. aureus* bacteremia was 2 times more likely to develop among Māori patients and 4 times more likely to develop among Pacific Peoples than among European patients (*7*). To date, however, studies describing *S. aureus* infections in New Zealand have generally been confined to 1 geographic region, to children, or to 1 specific aspect of *S. aureus* disease such as bloodstream or MRSA infection (*4*,*6*–*8*). Accordingly, we sought to describe the longitudinal trends for *S. aureus* infection and demographic characteristics of patients across the entire New Zealand population for the 12-year period 2000–2011.

## Methods

### Study Setting

New Zealand is an island nation in the southwestern Pacific Ocean and has ≈4.4 million residents. The population is ethnically diverse, consisting of the following ethnicities: 67% European, 15% Māori, 10% Asian, 7% Pacific Peoples, and 1% other (*10*). New Zealand has a public health care system; data on all publicly funded hospital admissions are recorded by the New Zealand Ministry of Health in the National Minimum Dataset (NMDS). In addition to basic patient information such as age, sex, and ethnicity, these data include principal and additional hospital discharge diagnoses, which since July 1999 have been coded according to the International Classification of Diseases, Tenth Revision (ICD-10). Our study population included all patients discharged from New Zealand hospitals from January 2000 through December 2011.

### Data Collection and Definitions

In New Zealand, a unique identifier (the National Health Index number) is assigned to each person who accesses public health services; this number can be used to extract information from the NMDS about patient hospitalizations. Patients were identified from the NMDS on the basis of *S. aureus*–associated ICD-10 discharge codes. These ICD-10 codes were A410 (sepsis due to *S. aureus*), J152 (pneumonia due to staphylococci), and B956 (*S. aureus* as the cause of diseases classified elsewhere). A case of *S. aureus* SSTI was defined as infection in a patient who had 1) a principal discharge diagnosis of SSTI (according to an epidemiologic case definition validated in a previous study [*11*]), 2) an additional discharge diagnosis of B956, and 3) no additional discharge diagnoses containing either A410 or J152. The National Health Index number can also be used to filter out unrelated hospital admissions. We filtered our data to exclude the following groups: overseas visitors, patients on waiting lists, hospital attendees who did not stay overnight, hospital transferees, and patients readmitted to the hospital within 30 days of first admission.

The following information about each patient who was discharged from the hospital for an *S. aureus*–associated cause was extracted from the NMDS: age, sex, ethnicity, and socioeconomic status (derived from the New Zealand deprivation index [NZDep] (*12*). The NZDep score is an area-based measure of socioeconomic deprivation derived from New Zealand census data; the score is based on various measures of deprivation, including household income, household ownership, household occupancy, employment and education levels, and access to telecommunications. It is expressed as a score between 1 and 10; a score of 10 represents the most deprived neighborhoods. To determine whether any increasing trends in *S. aureus* infection were associated with a general increase in all hospital admissions, we obtained information from the NMDS on all patients acutely hospitalized overnight in New Zealand over the study period, applying the same exclusion filters described above.

### Statistical Analyses

**Figure 1:**
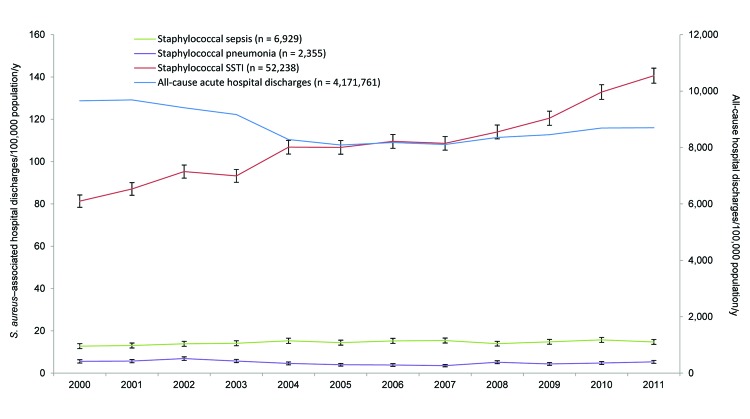
Annual rates of *Staphylococcus aureus*–associated hospital discharge (no. cases/100,000 population) and all-cause acute hospital discharge rates (no. cases/100,000 population), New Zealand, 2000–2011. Error bars indicate 95% CIs; for all-cause hospital discharges, error bars are too small to be visible on this chart. SSTI, skin and soft tissue infection.

Age-adjusted incidence rates were calculated per 100,000 population and standardized to the age distribution of the 2006 New Zealand census (*10*). These incidence rates were stratified according to sex, ethnicity, NZDep score, and geographic region. For analysis, we used 4 major ethnic groups: European, Māori, Pacific Peoples, and Asian/other. To determine possible geographic differences in incidence of *S. aureus* infection across New Zealand, we analyzed 4 broad geographic regions: northern, midland, central, and southern (Figure 1). Population denominator data were obtained from Statistics New Zealand (http://www.stats.govt.nz). A Poisson regression model, with log population data as an offset variable, was used to assess trends over time. The Kruskal-Wallis analysis of variance test was used to determine differences in the geographic incidence of *S. aureus* infections. Relative risks were calculated with 95% CIs, and all statistical analyses were performed by using SAS version 9.3 (SAS Institute Inc., Cary, NC, USA) or STATA version 11.1 (StataCorp, College Station, TX, USA). We considered p<0.05 to be statistically significant.

## Results

For the study period, 61,522 *S. aureus*–associated hospital discharges were identified. The overall averaged 12-year incidence rate for all *S. aureus* infections was 127 (95% CI 122–133) per 100,000 population per year. The overall incidence rate for *S. aureus* SSTIs was 108 (95% CI 105–111) per 100,000 population, *S. aureus* sepsis 14 (95% CI 13–16) cases per 100,000, and staphylococcal pneumonia 5 (95% CI 4–6) cases per 100,000. The incidence rate for sepsis caused by *S. aureus* and pneumonia caused by staphylococci did not change significantly over the study period; however, the incidence rate for *S. aureus* SSTIs increased significantly, from 81 (95% CI 78– 84) cases per 100,000 population in 2000 to 140 (95% CI 137–144) cases per 100,000 in 2011 (p<0.001) (Figure 1), which represents an increase of ≈5% each year. In contrast, the rate of acute all-cause hospital discharges in New Zealand fell significantly, from 9,657 (95% CI 9,625–9,689) per 100,000 population in 2000 to 8,701 (95% CI 8,673–8,729) per 100,000 population in 2011 (p<0.001). Consequently, the relative proportion of *S. aureus* SSTIs to all hospital discharges doubled, from 0.8% in 2000 to 1.6% in 2011.

**Figure 2:**
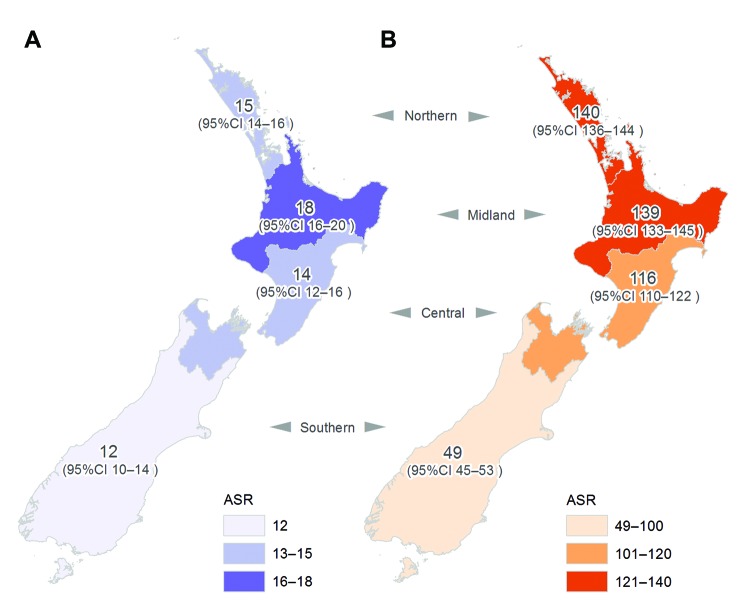
Average annual ASR (no. cases/100,000 population) of staphylococcal sepsis (A) and staphylococcal skin and soft tissue infections (B), New Zealand, 2000–2011. ASR, age-standardized rate.

Incidence of staphylococcal pneumonia did not vary significantly by geographic location (p = 0.8); however, incidence of staphylococcal sepsis (p = 0.02) and SSTIs (p = 0.01) did (Figure 2). In particular, there was a distinct north–south gradient for staphylococcal SSTIs; rates in the northern and central regions were ≈3 times rates in the southern region.

Incidence of *S. aureus* infections also varied markedly by sociodemographic characteristics (Appendix Table). Staphylococcal infections of all forms were significantly more likely to occur among male than female patients; this difference was most marked for *S. aureus* sepsis (relative risk [RR] 1.9; 95% CI 1.8–2.0). The incidence rates for sepsis and pneumonia were significantly higher among patients >70 years of age (62 and 24 cases/100,000 population/year, respectively) than among patients of other age groups (Appendix Table). In contrast, the incidence rate for *S. aureus* SSTIs was highest among those <5 years of age (242 cases/100,000 population/year). The incidence of all disease types was highest among Māori and Pacific Peoples (Appendix Table). In particular, Māori were 3 times more likely and Pacific Peoples almost 5 times more likely than Europeans to have an *S. aureus* SSTI. 

**Figure 3:**
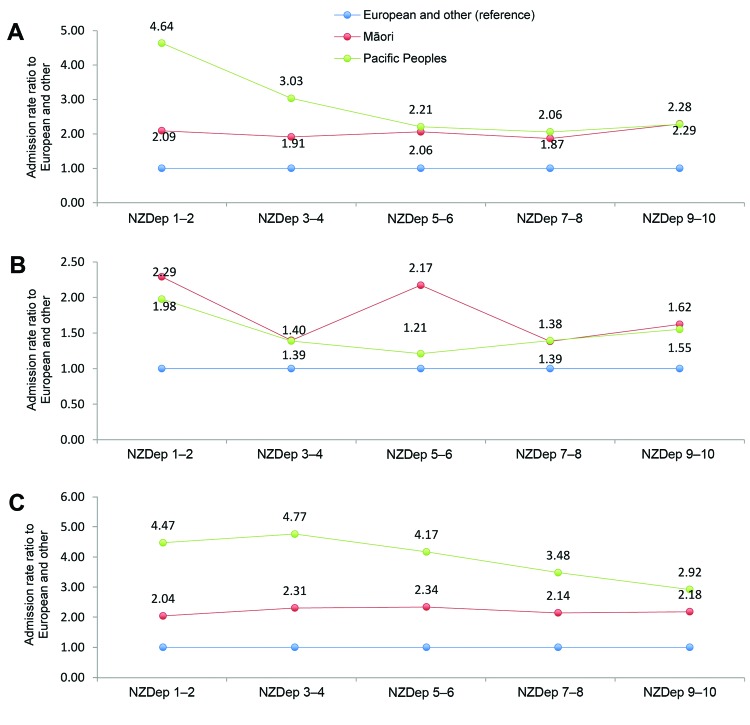
Admission rate ratios for *Staphylococcus aureus*–associated hospital discharges by ethnicity according to level of deprivation, New Zealand, 2000–2011. A) Staphylococcal sepsis; B) staphylococcal pneumonia; C) staphylococcal skin and soft tissue infections. NZDep, New Zealand Deprivation Index.

The incidence of *S. aureus* disease also varied significantly according to socioeconomic deprivation; the incidence rates for sepsis, pneumonia, and SSTI were significantly higher among patients residing in areas of high socioeconomic deprivation. This disparity was most marked for SSTIs; patients residing in areas of high deprivation were almost 4 times more likely to have *S. aureus* SSTIs than were those residing in areas of low deprivation (RR 3.7, 95% CI 3.6–3.8). An independent association seemed to exist between *S. aureus* disease and ethnicity after socioeconomic status was adjusted for, such that for each tier of socioeconomic deprivation, all 3 types of *S. aureus* disease were more common among Māori and Pacific Peoples than among those of European or other ethnicity (Figure 3).

## Discussion

In this study, we analyzed the longitudinal incidence and epidemiology of serious *S. aureus* disease across the entire New Zealand population during 2000–2011. Incidence of *S. aureus* SSTI increased dramatically while incidence of *S. aureus* sepsis and pneumonia remained relatively stable. Our finding of a persistent increase in serious *S. aureus* SSTIs over the past decade is a substantial public health concern, particularly given the overall decrease in acute overnight hospital admissions in New Zealand.

The factors underlying the increase in such infections are unknown, but risk factors for the development of *S. aureus* SSTI are multifactorial and probably include household crowding, delayed or inadequate access to health care, and issues associated with household hygiene (*13*,*14*). The reasons for the relatively unchanged rate of *S. aureus* sepsis and staphylococcal pneumonia in New Zealand are unclear; however, recent studies highlight the decreasing incidence of invasive *S. aureus* infections in other geographic settings, particularly among those patients recently exposed to health care facilities or receiving health care (*15*,*16*). Although improvements in infection prevention practices probably contribute to the decrease of invasive infections (*17*), other possible unexplored factors include changes in host susceptibility to *S. aureus* infection (e.g., improved management of concurrent conditions such as cardiovascular disease and diabetes) or temporal changes in the virulence profiles or transmissibility of circulating *S. aureus* strains.

Consistent with the findings of other studies of infectious diseases in New Zealand (*13*,*14*), we found notable sociodemographic disparity in the incidence of *S. aureus* infections; incidence of all *S. aureus* infections was highest among Māori or Pacific Peoples and among those residing in areas of high socioeconomic deprivation. Even after adjusting for socioeconomic deprivation, we found that the incidence of all *S. aureus* disease was significantly higher among Māori and Pacific Peoples than among patients of European and other ethnicities; this pattern is seen for infectious diseases generally in New Zealand (*14*). The underlying reasons for this apparent ethnic disparity in staphylococcal disease are uncertain. Unexplored possibilities include a higher prevalence of *S. aureus* colonization among Māori or Pacific Peoples or differences in the circulating *S. aureus* strain types among distinct ethnic groups, as previously described for our setting (*4*). However, an alternative possibility is that the area-based NZDep score used to record socioeconomic deprivation does not fully represent those facets of poverty that contribute to the development and prevention of serious *S. aureus* disease. These unmeasured risk factors include aspects of health literacy relating to early management of insect bites and skin infections, availability of household amenities such as hot water, and affordable and timely access to health care. Specific individual-level and household-level studies are required for determination of the relative contribution of such potentially modifiable risk factors.

We also observed significant geographic variation in the incidence of *S. aureus* SSTI, with a distinct north–south gradient. This finding can probably be explained by the distribution of population groups in New Zealand; the groups most affected by *S. aureus* SSTI reside predominantly in the North Island (*13*). However, other possible contributory factors include geographic differences in access to and provision of health care and climate differences; the climate in the upper North Island is relatively warmer and more humid than that in the southern regions.

A limitation of our study was our use of hospital discharge data for case ascertainment. Use of these data meant that we were unable to determine the proportion of cases occurring in the community versus in the hospital setting, although previous studies have demonstrated that most *S. aureus* infections in New Zealand originate in the community (*4*,*7*,*8*). However, our aim was not to provide detailed information on individual *S. aureus* infections but rather to provide a broad overview of the trends and demographics of serious *S. aureus* infections across the entire New Zealand population. In addition, our data represent only those patients whose hospital discharge was associated with *S. aureus* disease; they do not represent those patients who sought care from a primary care physician or who sought care at a hospital but were not admitted. For example, a recent study of children with SSTIs in 1 New Zealand region found an estimated 14 primary care cases for every 1 hospital admission (*18*). Furthermore, these data represent only those instances in which an etiologic agent was described and recorded in the discharge diagnoses. It is therefore highly likely that the overall prevalence of staphylococcal disease in our setting is substantially higher than that estimated here.

In summary, our study provides valuable longitudinal data on the prevalence of serious *S. aureus* disease in the New Zealand population and represents one of the few studies that systematically assessed the epidemiology and demographics of staphylococcal infections across an entire nation. The steady and significant increase in serious *S. aureus* SSTI coupled with notable sociodemographic disparity in disease incidence is a disturbing national trend. A concerted multimodal public health intervention is urgently required to tackle this problem.
